# Sestrin2 as a Novel Biomarker and Therapeutic Target for Various Diseases

**DOI:** 10.1155/2017/3296294

**Published:** 2017-06-11

**Authors:** Mazhar Pasha, Ali H. Eid, Assaad A. Eid, Yves Gorin, Shankar Munusamy

**Affiliations:** ^1^College of Pharmacy, Qatar University, P.O. Box 2713, Doha, Qatar; ^2^Department of Pharmacology and Toxicology, Faculty of Medicine, American University of Beirut, Beirut, Lebanon; ^3^Department of Biological and Environmental Sciences, College of Arts and Sciences, Qatar University, P.O. Box 2713, Doha, Qatar; ^4^Department of Anatomy, Cell Biology and Physiological Sciences, Faculty of Medicine, American University of Beirut, Beirut, Lebanon; ^5^School of Medicine, University of Texas Health Sciences Center at San Antonio, San Antonio, TX, USA

## Abstract

Sestrin2 (SESN2), a highly conserved stress-inducible metabolic protein, is known to repress reactive oxygen species (ROS) and provide cytoprotection against various noxious stimuli including genotoxic and oxidative stress, endoplasmic reticulum (ER) stress, and hypoxia. Studies demonstrate that the upregulation of Sestrin2 under conditions of oxidative stress augments autophagy-directed degradation of Kelch-like ECH-associated protein 1 (Keap1), which targets and breaks down nuclear erythroid-related factor 2 (Nrf2), a key regulator of various antioxidant genes. Moreover, ER stress and hypoxia are shown to induce Sestrins, which ultimately reduce cellular ROS levels. Sestrin2 also plays a pivotal role in metabolic regulation through activation of the key energy sensor AMP-dependent protein kinase (AMPK) and inhibition of mammalian target of rapamycin complex 1 (mTORC1). Other downstream effects of Sestrins include autophagy activation, antiapoptotic effects in normal cells, and proapoptotic effects in cancer cells. As perturbations in the aforementioned pathways are well documented in multiple diseases, Sestrin2 might serve as a potential therapeutic target for various diseases. Thus, the aim of this review is to discuss the upstream regulators and the downstream effectors of Sestrins and to highlight the significance of Sestrin2 as a biomarker and a therapeutic target in diseases such as metabolic disorders, cardiovascular and neurodegenerative diseases, and cancer.

## 1. Introduction

Sestrins (SESN) are highly conserved proteins with pleiotropic biological functions and are upregulated in cells under stressful conditions such as DNA damage, hypoxia, starvation, growth factor depletion, radiation, and oxidative stress [1, 2]. Following their induction, Sestrins protect cells against genotoxic and oxidative stress and are, thus, named as stress-inducible metabolic regulators [1]. Sestrin proteins comprise of three distinct family members, characterized by specific protein-coding genes such as SESN1, SESN2, and SESN3, which share nearly 50% identical amino acid sequences [1, 3]. Despite their genetic homology, identification of the specific biochemical functions of each Sestrin was challenging, as their protein structure contains an atypical structural domain. The distant sequence homology of Sestrins to bacterial oxidoreductases led to the discovery of their antioxidant properties in mammalian cells [1]. SESN1, also known as p53-activated gene 26 (PA26) because it is regulated by tumor-suppressor protein (p53), has been recognized as one of the growth arrest and DNA damage-inducible genes (GADD) [3]. SESN2, a homolog of PA26, is referred to as hypoxia-inducible gene 95 (Hi95), owing to its induction under hypoxic conditions, albeit other cytotoxic events such as oxidative stress and DNA damage also induce SESN2 levels [3–5]. SESN3 is as a novel PA26 structure-related gene inducible by the forkhead box O (FoxO) family of transcription factors [6].

## 2. Sestrins: Isoforms, Their Regulation, and Cellular Effects

As stress-inducible metabolic regulators, Sestrins help cells to adapt to various stress stimuli via multiple mechanisms including activation of catabolic reactions, cessation of anabolic activities, and initiation of cell repair mechanisms to maintain cellular homeostasis [1]. Specifically, Sestrins play a cytoprotective role via decreasing the levels of reactive oxygen species (ROS) resulting from oxidative and genotoxic stress [3]. The tumor suppressive p53 gene, which regulates many stress-activated transcriptional factors, is critical for the expression of Sestrin1 following oxidative stress [1]. In addition to p53, the nuclear factor (erythroid-derived 2-) like 2 (Nrf2) and activator protein 1 (AP-1) are requisite for Sestrin2 stimulation; correspondingly, the AP-1 and the forkhead box O (FoxO) transcriptional proteins FoxO1 and FoxO3 are essential for Sestrin3 induction [6, 7], as shown in Table 1. Moreover, the inhibition of ROS by Sestrins might be elicited directly through their enzymatic activity (peroxiredoxin) or by activation of Nrf2, a master key regulator of various antioxidant genes [5, 7]. Therefore, Sestrins prevent the accumulation of ROS through its antioxidant properties via multiple signaling pathways.

Parallel to its redox-regulating actions, Sestrins inhibit mammalian target of rapamycin complex 1 (mTORC1) activity primarily through the activation of adenosine monophosphate-dependent protein kinase (AMPK) [1, 6, 21]. The activation of AMPK by Sestrins might be via direct physical association or by indirect transcriptional regulation. Apart from its actions mediated through AMPK, Sestrins act as inhibitors of GTPase-activating protein for Rag (Rag GTPases), which are important for mTORC1 activity. Through these concerted actions, Sestrins inhibit mTORC1 and consequently reduce protein synthesis during unfolded protein response (UPR) and protect cells against endoplasmic reticulum (ER) stress [22].

Sestrins also play a vital role in metabolic homeostasis via upstream regulation of the mTORC1 and AMPK signaling pathways, which are critical for energy and nutrient sensing in cells. Inactivation of Sestrin genes result in various cellular and metabolic pathologies, such as oxidative damage, mitochondrial dysfunction, fat accumulation, muscle degeneration, and accelerated progression of diabetic complications [23–25]. Hence, it is critical to understand the role of Sestrins in the modulation of pathophysiologic mechanisms such as oxidative stress, autophagy, ER stress, apoptosis, and hypoxia, which are closely linked to altered mTOR/AMPK signaling in cells.

## 3. Upstream Pathways and Mechanisms that Modulate Sestrins

### 3.1. Oxidative Stress

Oxidative stress occurs when there is an imbalance between the generation of reactive oxygen species (ROS) and reactive nitrogen species (RNS) and their scavenging mechanisms. Sestrin family of proteins is one of the several antioxidant defense mechanisms, which primarily gets stimulated under oxidative stress, although other noxious stimuli could induce Sestrins [5, 26, 27]. High levels of ROS stimulate a series of antioxidant genes mainly through activation of the antioxidant response elements (AREs) [28]. Nuclear factor (erythroid-derived 2-) like 2 or NF-E2-related factor-2 (Nrf2) is an important transcriptional factor (from the family of basic leucine zipper (bZIP) proteins) that regulates the expression of various antioxidant genes through binding to AREs [7, 28]. Under normal conditions, Nrf2 is localized in cytosol with its repressor Kelch-like ECH-associated protein 1 (Keap1) and subjected to ubiquitination and proteasomal degradation via Cul3-based E3 ligase. Oxidative stress dissociates the repressor molecule Keap1 from Nrf2, and facilitates the nuclear translocation of Nrf2 and its binding and activation of AREs [7]. Nrf2 is ubiquitously expressed in mammalian cells and plays an essential role as a cytoprotector under severe stress-related conditions [28].

A study by Shin et al. [28] demonstrated the central role of Nrf2-ARE system in the regulation of Sestrin2 expression. Specifically, the study revealed that Nrf2 activators such as tert-butylhydroquinone and sulforaphane upregulate the expression of Sestrin2 mRNA in a dose- and time-dependent manner. The mechanism by which Sestrin2 activates Nrf2 expression was unveiled in a study by Bae et al. [7]. The study revealed that the antioxidant effects of Sestrin2 are mediated by the degradation of Keap1 through p62-dependent autophagy and consequent activation of Nrf2. Furthermore, a study by Kim et al. [29] demonstrated that the injection with Ad-SESN2 (recombinant adenovirus encoding Sestrin2) in mice not only inhibits acetaminophen-induced oxidative stress and inflammatory response but also prevents acetaminophen-induced liver toxicity and associated mortality.

**Figure 1:**
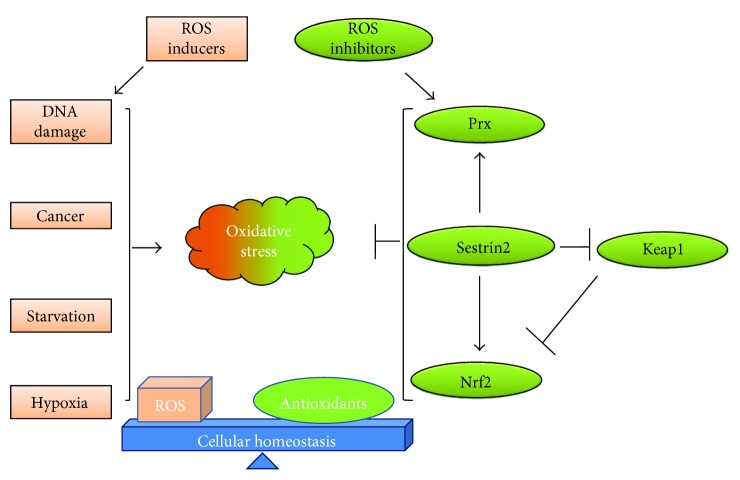
Role of Sestrin2 in redox balance. Cellular homeostasis is achieved by counteracting the production of reactive oxygen species (ROS) with antioxidant molecules. Hypoxia, starvation, cancer, and genotoxic stress induce oxidative stress in cells and tip the cellular redox balance towards a pro-oxidant state. Sestrins help cells to restore their normal redox state through various mechanisms including stabilization of Nrf2 via the inhibition of Keap1 and recycling of peroxiredoxin (Prx). Keap1: Kelch-like ECH-associated protein 1; Nrf2: nuclear factor (erythroid-derived 2-) like 2.

Cancer cells, unlike normal cells, favor conditions of oxidative stress and fuel ROS to support their high cell proliferation and to promote mutations, ultimately resulting in genomic instability and cell survival. Despite the high levels of ROS, most forms of cancer are associated with significant downregulation of Sestrin2 [4, 12, 22]. Conversely, the induction of Sestrin2 in various cancer cell lines has been shown to curb oxidative stress and slow tumorigenesis [3, 4, 11]. Together, these findings underscore that the antioxidant effects of Sestrin2 confer cytoprotection, prevent organ damage, and abrogate tumorigenesis as shown in Figure 1().

### 3.2. Endoplasmic Reticulum (ER) Stress

Endoplasmic reticulum (ER) stress occurs when unfolded proteins get accumulated within the lumen of ER due to adverse physiologic conditions. The three ER transmembrane enzymes, protein kinase RNA-like endoplasmic reticulum kinase (PERK), inositol-requiring enzyme 1 (IRE1), and activating transcription factor 6 (ATF6), which monitor the health of ER, play a crucial role in the remediation of ER stress via triggering an unfolded protein response (UPR) [30]. The UPR is recognized as an integrated signal transduction mechanism, whose primary objective is to restore ER homeostasis via diverse but interconnected mechanisms. All three pathways, PERK, IRE1, and ATF6, ultimately evoke an adaptive response through upregulation of the key ER chaperones, repression of protein translation, and stimulation of protein degradation by ER-associated degradation (ERAD) mechanism. When ER stress continues for prolong periods, the UPR machinery preferentially stimulates apoptotic pathways and leads to cell death [30, 31].

Several studies have demonstrated the upregulation of Sestrin2 under ER stress conditions [30–33]; however, the exact mechanism by which ER stress induces Sestrin2 expression is poorly understood. For instance, Park et al. [31] demonstrated that the induction of Sestrin2 in response to palmitate-induced ER stress in hepatocytes occurs through PERK-c/EBP*β*-mediated signal transduction mechanism. Consistent with these findings, a study in cancer cells by Bruning et al. [32] showed that the induction of Sestrin2 is dependent on the PERK via activating transcription factor 4 (ATF4). A recent study by Saveljeva et al. [33] revealed that mouse embryo fibroblasts (MEFs) deficient in PERK or X-box-binding protein-1 (XBP1, a downstream marker of IRE1) are unable to induce Sestrin2 expression in response to ER stress. Similarly, knockdown of XBP1 in cancer cell lines—HCC1806 and MCF7—prevented Sestrin2 induction that occurs in response to thapsigargin- and methotrexate-mediated ER stress. Together, these studies indicate that the upregulation of Sestrin2 under ER stress is dependent on both PERK and IRE1/XBP1 transduction pathways [33].

**Figure 2:**
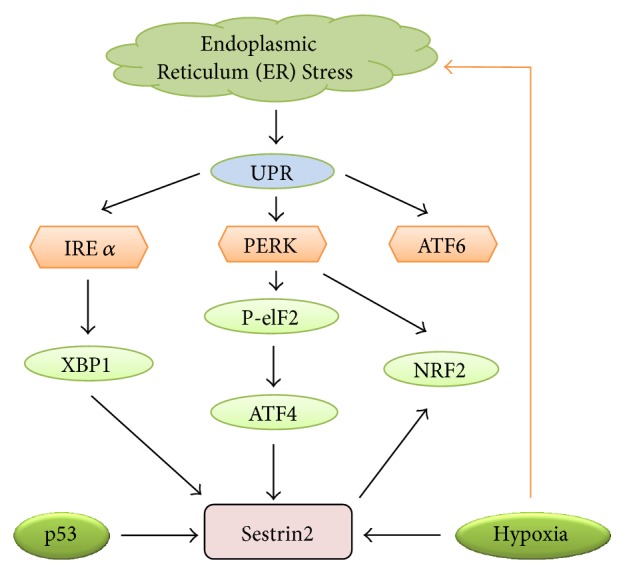
Upregulation of Sestrin2 under endoplasmic reticulum stress, genotoxic stress, and hypoxia. UPR mediators play a significant role in ER stress-mediated upregulation of Sestrin2. Two of the three UPR mediators, IRE1 (via XBP1) and PERK (through P-eIF2 and Nrf2), upregulate Sestrin2. Similarly, independent of ER stress, p53 (in response to genotoxic stress) and hypoxia also activate Sestrin2 expression. ATF4: activating transcription factor 4; ATF6: activating transcription factor 6; IRE1: inositol-requiring enzyme 1; NRF2: nuclear factor (erythroid-derived 2-) like 2; P-eIF2: phosphorylated eukaryotic initiation factor 2; PERK: protein kinase RNA-like endoplasmic reticulum kinase; UPR: unfolded protein response; XBP1: X-box-binding protein-1.

Recently, a study by Ding et al. [30] demonstrated the upregulation of Sestrin2 expression through ATF4 and Nrf2 transcription factor under ER stress induced by glucose starvation. The study further demonstrated that both transcription factors (ATF4 and Nrf2) get stimulated through the PERK1 pathway of UPR and directly bind to Sestrin2 promoter. Interestingly, the induction of Sestrin2 by ATF4 and Nrf2 occurs through a mechanism that is independent of p53, the master regulator of Sestrin2 induction upon DNA damage. Hence, it could be proposed that the activation of Sestrin2 occurs via two mechanisms: first, through p53 in response to DNA damage and, second, by UPR-mediated activation of ATF4 and Nrf2 [30]. The ER stress pathways that upregulate Sestrin2 are shown in the Figure 2().

### 3.3. Hypoxia

Low-oxygen tension or hypoxia is one of the prominent stimuli that is known to activate the expression of Sestrin2. In fact, Sestrin2 was first isolated as a gene activated in human neuroblastoma cells under hypoxia conditions [5, 9]. For example, exposure of cancer cells to hypoxia upregulates both Sestrin1 and Sestrin2. However, unlike Sestrin1, the activation of Sestrin2 is independent of p53 and is mainly due to energy deprivation secondary to prolonged hypoxia [1, 5]. Nevertheless, some exceptions do exist as Sestrin2 could be activated via mechanisms dependent [5] as well as independent [34] of hypoxia-inducible factor (HIF-1) as shown in the Figure 2(). Moreover, the mechanism by which certain drugs and chemicals such as metformin and 2-deoxyglucose, which stimulate hypoxia, activate Sestrin2 is yet to be deciphered [35].

Shi et al. [17] studied the relationship between HIF-1*α* protein and recombinant human Sestrin2 in both severe and moderate hypoxic-ischemic (HI) injuries in neonatal rats. Data from the study suggests a distinct and profound neuronal upregulation of Sestrin2 by HIF-1*α* under severe HI injury as compared to moderate HI injury. In addition, the HIF-1*α*-mediated induction of Sestrin2 in neurons was demonstrated to attenuate blood-brain barrier (BBB) permeability through suppression of vascular endothelial growth factor (VEGF) in neonatal rats subjected to severe HI injury [17]. Similarly, Sestrin2 activation was shown to inhibit angiogenesis and VEGF production in a cancer xenograft model [18]. Furthermore, the hypoxia-mediated upregulation of Sestrin2 also serves to counteract ROS generation in cells. This evidence was revealed in a study by Essler et al. [36], which evaluated the significance of hypoxia and nitric oxide (NO) on the transcription regulation of various genes involved in peroxide signaling pathway using RAW 264.7 cells (a macrophage cell line). Findings from the study demonstrate that hypoxia and NO upregulate Sestrin2 in a HIF-1*α*-dependent manner, and the resultant activated Sestrin2 augments peroxide defense by preventing peroxiredoxins from sulfinylation [36]. Therefore, Sestrin2 not only serves as a molecule to integrate the signals of hypoxia and oxidative stress but also acts as a defense mechanism to combat against cellular stress.

## 4. Downstream Pathways and Mechanisms Modulated by Sestrins

### 4.1. AMP-Activated Protein Kinase (AMPK) Activation and mTORC1 Inhibition

The mechanistic/mammalian target of rapamycin (mTOR) is a serine-threonine protein kinase initially discovered in yeast mutants that are resistant to the growth inhibitory actions of rapamycin and was soon after cloned in mammalian cells. mTOR comprises of two distinct multiprotein complexes: the mechanistic target of rapamycin complex 1 (mTORC1), which is sensitive to rapamycin, and the mTORC2, which is insensitive to rapamycin. The mTORC1 protein kinase is regarded as a “master regulator” as it responds to various stimuli including growth factors, oxidative stress, and alterations in energy levels [37, 38]. Stimulation of mTORC1 leads to phosphorylation of two proteins, p70 ribosomal protein S6 kinase (p70S6K) and 4E-binding protein-1 (4EBP1), and ultimately results in increased protein and lipid synthesis, cell proliferation, and/or survival [37, 38]. Persistent mTOR stimulation is linked to various diseases such as diabetes, obesity, cardiovascular diseases, cancer, and autoimmune disorders [38].

AMP-activated protein kinase (AMPK), an enzyme activated under the conditions of energy deficiency, serves as the principal negative regulator of mTOR in cells. Studies indicate that Sestrin2 inhibits mTOR activation in cells mainly through the activation of AMPK and phosphorylation of tuberous sclerosis 2 (TSC2) [9, 23]. Moreover, genetic silencing and knockdown of Sestrin2 in vitro and in vivo cause sustained activation of mTOR signaling in multiple cell types including liver, indicating the essential role of Sestrin2 on mTOR inhibition. For instance, a study by Hamatani et al. [39] demonstrated that silencing of Sestrin2 upregulates the phosphorylation of mTORC1 downstream targets such as p70S6K in parietal epithelial cells. Intriguingly, chronic activation of mTOR due to overnutrition in mice eventually leads to the induction of Sestrin2 in multiple tissues including liver and skeletal muscle [23]. Conversely, genetic ablation of Sestrin2 augments mTOR activation and aggravates obesity-associated features such as glucose intolerance, insulin resistance, and hepatosteatosis in mice [23]. Consistent with the above findings, the inhibition of AMPK using compound C was shown to upregulate Sestrin2 via induction and accumulation of mitochondrial ROS [40].

**Figure 3:**
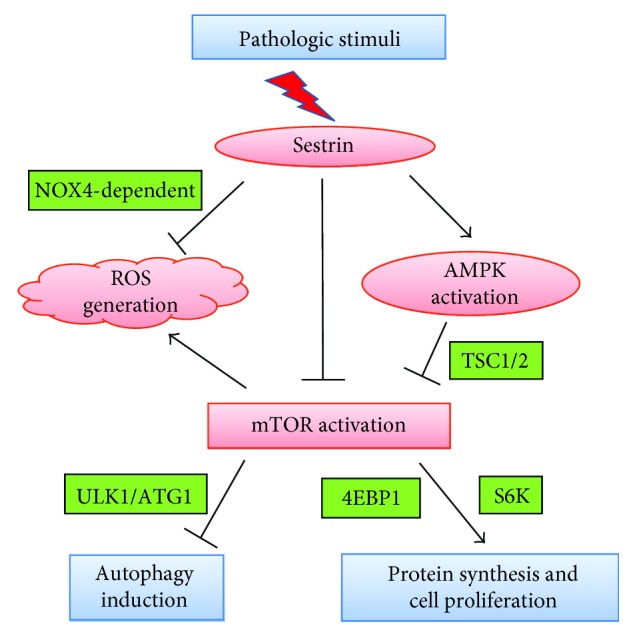
Modulation of AMPK and mTOR pathway by Sestrins. Activation of mTOR signaling induces reactive oxygen species (ROS) generation, inhibits autophagy, and promotes protein synthesis and cell proliferation. Sestrins downregulate mTOR signaling pathway and activate AMPK signaling, which in turn relieves autophagy inhibition and reduces protein synthesis and cell proliferation. In addition, Sestrins decrease ROS generation through the inhibition of NAPDH oxidase 4- (NOX4-) dependent ROS generation pathway. 4EBP1: eukaryotic translation initiation factor 4E-binding protein 1; ATG1: autophagy-related protein 1; S6K: ribosomal protein S6 kinase; TSC1/2: tuberous sclerosis complex 1 and 2; ULK1: unc-51-like autophagy-activating kinase 1.

An inverse modulation of mTOR and AMPK signaling by Sestrin2 appears to be responsible for its neuroprotective effects. For example, Shi et al. [41] revealed that the neuroprotective effects of Sestrin2 following hypoxic-ischemic encephalopathy are mediated through modulation of AMPK and mTOR signaling pathway. In addition, the study also demonstrated that administration of recombinant human Sestrin2 significantly enhances neurological function besides reducing cerebral infarction and brain atrophy. The critical role of AMPK activation on the antioxidant properties of Sestrin2 was revealed in a study by Eid et al. [21]. The study demonstrated that Sestrin2-mediated activation of AMPK pathway also inhibits NADPH-dependent oxidase 4 (NOX4), consequently, reducing cytosolic ROS produced by NOX4 oxidase system [21]. The pathways that interconnect the regulation of ROS and AMPK signaling pathways and their downstream mediators are depicted in the Figure 3.

### 4.2. Autophagy

Autophagy is one of the cellular defense mechanisms that directs aged and misfolded proteins for lysosomal degradation and thereby protects cells from undue stress and maladaptive cell signaling [42, 43]. A major stimulus for autophagy activation in cells is the inhibition of mTOR signaling and/or activation of AMPK through phosphorylation of autophagy-related protein (ATG) and unc-51-like autophagy-activating kinase 1 (Ulk1) protein [44]. As described earlier, Sestrin2 regulates mTORC1/AMPK signaling pathway; accordingly, it is expected that the activation of Sestrin2 would stimulate autophagy in cells. Indeed, Sestrin2, in concerted action with several autophagy-related proteins (ATG) and BCL2/adenovirus E1B 19 kDa protein-interacting protein 3 (BNIP3), has been shown to induce autophagy [45]. Moreover, the inhibition of mTORC1 by Sestrin2 is important for the p62 autophagy-mediated breakdown of Kelch-like ECH-associated protein 1 (Keap1), the repressor molecule that directs Nrf2 to proteasomal degradation [7]. Thus, autophagy activation is an essential requirement for the antioxidant effects of Sestrin2—mediated by Nrf2 signaling—to prevail during oxidative stress.

Furthermore, studies in cancer cells [10, 46] reveal that the induction of autophagy by Sestrin2 is regulated by c-Jun N-terminal kinase (JNK) pathway. Studies in nasopharyngeal carcinoma cell lines by Zhang et al. [10] demonstrated that stimulation of JNK pathway increases the expression of Sestrin2, and this induction of Sestrin2 could be attenuated by siRNA-mediated silencing of JNK pathway. Interestingly, the JNK-mediated autophagy was inhibited following silencing of the expression of Sestrin2, which indicates that the JNK-mediated autophagy induction in cancer cells occurs in a Sestrin2-dependent manner. A recent study by Liang et al. [46] in human bladder cancers unveiled the mechanism by which JNK activates Sestrin2 and stimulates autophagy in cancer cells. Findings from this study indicate that binding of JUN (resulting from activation of JNK pathway) to the AP-1 binding site in the Sestrin2 promoter region is critical for the induction of Sestrin2 and consequent autophagy activation in cancer cells. Taken together, Sestrin2 plays an important role in the stimulation of autophagy [10, 46] and, hence, serves as a mediator for cells to integrate the nutrient signals (AMPK and mTOR) and signaling pathways such as JNK to modulate autophagic response and promote the survival of normal but the death of cancer cells.

### 4.3. Apoptosis

Programmed cell death, commonly referred to as apoptosis, serves as a process to control cell growth and development. Apoptosis is characterized by specific biochemical and morphological changes including membrane blebbing, cell shrinkage, nuclear condensation, chromosomal DNA fragmentation, and cleavage of intracellular proteins by specific enzymes called caspases [39]. Numerous studies have documented the antiapoptotic role of Sestrin2 in various cell types [41, 47]. A study in RAW264.7 cells by Hu et al. [47] reported that exposure to oxidized low-density lipoprotein (oxLDL) stimulates the expression of Sestrin2 through JNK/c-Jun pathway and knockdown of Sestrin2 promotes apoptosis. In correlation to these findings in vitro, intranasal administration of recombinant human Sestrin2 in neonatal rats subjected to hypoxic-ischemic injury was shown to reduce neuronal apoptosis and improve neurological function [41]. Thus, induction of Sestrin2 serves as a compensatory response to attenuate apoptosis induced by various noxious stimuli. In contrast, downregulation of Sestrin1/2 in cancerous cells causes accelerated tumor cell growth [11, 46], which indicates that Sestrins elicit proapoptotic effects in cancer cells. Thus, Sestrin2 could serve as a biomarker and a therapeutic target in various diseases such as cardiovascular and metabolic disorders, neurodegenerative diseases, and cancer.

## 5. Significance of Sestrins in Diseases

Based on the mounting evidences from the literature, it is evident that Sestrins get upregulated in response to stress stimuli (such as oxidative stress, genotoxic stress, ER stress, and hypoxia), and they exert cytoprotective actions via modulation of various cell signaling processes including nutrient sensing, autophagy, and apoptosis. Hence, Sestrins are implicated to play a protective role in various diseases including cardiovascular and metabolic disorders, neurodegenerative diseases, and cancer.

### 5.1. Significance of Sestrins in Cardiovascular Diseases

Increased cardiac load or damage leads to cardiac hypertrophy, which ultimately contributes to the malnutrition and death of cardiomyocytes. Indeed, cardiac hypertrophy is one predictor of mortality and morbidity associated with cardiovascular diseases. Sestrins appear to play a role in augmenting the antioxidant status within cells [5]. Because oxidative stress is a major player in cardiac pathophysiology, it was expected that Sestrins may be protective in cardiomyopathies. Mechanistically, it was found out that loss of Sestrin results in mTOR hyperactivation, associated with decreased cardiac function, and that inhibition of mTOR rescues the deranged phenotype resulting from Sestrin downregulation [14]. This is in line with earlier studies showing that Sestrin1 effectively suppresses angiotensin II-induced proliferation of cardiac fibroblasts by its ability to inhibit mTOR signaling [14]. Indeed, inhibiting mTOR appears to be cardioprotective especially under stressful conditions such as those resulting from pressure overload [15, 48].

Another mechanism by which Sestrin appears to affect cardiac function is via its modulation of autophagic pathways. Tight regulation of autophagy may be beneficial in protecting against pressure overload cardiac hypertrophy [49]. On the contrary, blocking autophagy may further aggravate the hypertrophic phenotype [16]. Knockdown of Sestrin1 exacerbates phenylephrine-induced hypertrophy of cardiomyocytes, whereas its overexpression protects these cells from hypertrophic stress [16]. Sestrin1 appears to elicit this effect via activating the AMPK/mTORC1/autophagy axis, and blockade of this pathway has been shown to reduce the Sestrin's ability to protect against hypertrophic stressors [16]. Furthermore, the activation of AMPK through its upstream kinase liver kinase B1 (LKB1) was demonstrated to be essential for the maintenance and enhancement of autophagic activity by Sestrin2 [8]. Consequently, the activation of AMPK controls mitochondrial biogenesis and conserves the energy balance and, eventually, helps cells to survive under ischemic conditions.

Sestrins also appear to play a beneficial role in atherosclerosis. Indeed, it was recently shown that knockdown of Sestrin2 potentiates the formation of atherosclerotic plaques and other hallmarks of atherosclerosis in mice [50]. The underlying mechanism for this effect appears to involve increased ROS production and ER stress as well as increased adhesion molecules in the vasculature [50]. Taken together, Sestrins 1 and 2 may prove to be attractive targets for amelioration of cardiovascular diseases.

### 5.2. Significance of Sestrins in Metabolic Disorders

Metabolic disorders such as diabetes and obesity are marked by alterations in the principal nutrient-sensing mechanisms AMPK and mTOR and are associated with an increased risk of cardiovascular diseases and stroke [21, 37, 38]. Chronic activation of mTORC1 during overnutrition increases protein and lipid synthesis and represses autophagic catabolism in cells [23, 51]. One of the prominent negative feedback mechanisms to circumvent the harmful effects of chronic mTORC1 activation is the transcriptional activation of Sestrin2. Upon activation, Sestrin2 activates AMPK signaling, which in turn attenuates the activation of mTORC1 and, thus, secondarily stimulates autophagy in cells [9, 23]. For example, a study by Lee et al. [23] demonstrated that Sestrin2 was upregulated in various tissues such as muscle, liver, and adipose tissues in a mice model of type 2 diabetes and obesity. Similarly, Kimball et al. [52] also showed the upregulation of Sestrin2 expression in the livers of rats fed with a high-fat diet.

Chronic mTORC1 stimulation along with persistent inhibition of autophagy in hepatocytes leads to insulin resistance and type 2 diabetes mainly via inhibition of the phosphorylation of insulin receptor substrates (IRSs) [53]. Chai et al. [25], in a study, demonstrated that insulin upregulates Sestrin2 in mouse primary hepatic cells and hepatic tumor cell lines. The upregulation of Sestrin2 expression by insulin was demonstrated to be mediated through PI3K/PKB/mTOR signaling pathway. Thus, it is evident that a molecular loop exists between insulin and Sestrin2 during chronic activation of mTORC1, and further studies to understand the intricate feedback that exists between insulin and Sestrin2 would give us insights to develop novel therapeutic strategies to treat metabolic disorders [25].

### 5.3. Significance of Sestrins in Neurodegenerative Disorders

Increased ROS levels damage neurons by overwhelming the natural antioxidant defense mechanism, which ultimately induces apoptosis. Thus, one of the effective strategies to treat neurodegenerative diseases such as Parkinson's disease (PD) and Alzheimer's disease (AD) is to protect neuronal cells from oxidative injury. Since Sestrin2 inhibits the generation and accumulation of ROS, its effects are most useful in the prevention and treatment of neurodegeneration and various age-related disorders [19, 24].

The ability of Sestrin2 to inhibit mTORC1 and indirectly activate autophagy is another mechanism that is most useful in treating neurodegenerative diseases. For instance, dysregulation of the autophagy-lysosomal pathway is one of the key mechanisms responsible for the accumulation of *α*-synuclein, a major component of Lewy bodies observed in the brain of patients with PD. Using an in vitro model of PD, Hou et al. [19] showed that the upregulation of Sestrin2 triggers autophagic response and subsequently prevents *α*-synuclein expression, apoptotic caspase-3 activation, and cytotoxicity in dopaminergic cells. Intriguingly, the observed neuroprotection was attenuated following siRNA-mediated genetic silencing of Sestrin2 expression, which indicates the protective role of Sestrin2 against neurodegeneration. In corroboration to these findings, Zhou et al. [54] reported the upregulation of Sestrin2 in the midbrain of patients with PD. Additional in vitro studies using 1-methyl-4-phenylpyridinium- (MPP+-) induced neurotoxicity in human neuroblastoma SH-SY5Y cells by the same investigators unveiled an induction of Sestrin2 mRNA and protein levels via activation of p53-mediated pathway. Moreover, knockdown of Sestrin2 intensified MPP+-induced neurotoxicity.

Similar to the findings obtained from the experimental models of PD, using an in vitro model of AD, Chen et al. [20] identified Sestrin2 as one of the target genes that was induced upon exposure to amyloid beta (A*β*) peptide in human neuroblastoma cells. Knockdown of Sestrin2 was shown to abrogate autophagic response and aggravate A*β*-induced neurotoxicity. Although the exact mechanisms by which Sestrin2 modulates autophagic response and suppresses A*β*-induced neurotoxicity in AD is yet to be evaluated, the defensive role of Sestrin2 against neurodegeneration makes it a positive prognostic marker and a pharmacological target in neurodegenerative diseases.

### 5.4. Significance of Sestrins in Cancer

Increased production and accumulation of ROS and the resultant alterations in cellular redox status in tumor cells favors genomic instability and allows subsequent mutations to support tumor progression [55]. Moreover, in an advanced stage, cancer cells recurrently show high levels of ROS, which facilitates tumor metastasis [55]. Thus, inhibition of ROS generation might serve as a viable strategy to inhibit the progression of cancer. Sestrin2, a novel p53-dependent stress-inducible protein, which gets activated under hypoxic conditions, is downregulated in cancer cells [11, 46]. Similarly, deficiency of Sestrin2 in mouse embryonic fibroblasts was associated with the increased expression of cyclin D1 (a cell cycle regulator) and increased Ras-activated tumor cell growth compared to wild type cells [9].

Most cancer cells induce the expression of HIF-1*α* for their survival in a hypoxic tumor microenvironment [55]. Thus, inhibition of HIF-1*α* expression is deemed as an effective strategy to inhibit the progression and metastases of tumor cells. In this regard, a study by Seo et al. [4] demonstrated that the overexpression of Sestrin2 represses the accumulation of HIF-1*α* (a key transcription factor involved in tumorigenesis and hypoxia-dependent gene transcription) and prevents the metastasis of colorectal cancer. Besides, the study also revealed the involvement of AMPK in Sestrin2-mediated degradation of HIF-1*α* through the upregulation of prolyl-4-hydroxylases (PHD) and targeted hydroxylation of HIF-1*α*. Furthermore, knockdown of AMPK via siRNA prevented the inhibitory actions of Sestrin2 on HIF-1*α* accumulation. Collectively, it could be concluded that Sestrin2 acts as a negative regulator of tumor progression via an AMPK-dependent mechanism.

The significance of mTOR hyperactivation leading to tumorigenesis and tumor progression is well documented [37]. mTOR activation-induced protein synthesis also leads to unfolded protein response, which if unresolved, results in sustained ER stress and ultimately causes tissue injury [31]. Thus, as a negative regulator of mTOR, it is expected that Sestrin2 could serve as a biomarker and a therapeutic target in cancer. In support to this notion, a study by Ro et al. [22] demonstrated that treatment with 5-fluorouracil (5FU) induces the expression of Sestrin2, which in turn represses the in vitro migration of colon cancer cell lines such as HCT116 and HT29 cells. Recent studies by Seo et al. [56] in colon cancer cells also reveal that 5FU-mediated induction of Sestrin2 occurs through p53-dependent pathway.

Growing body of evidences from numerous cancer cell lines highlights the role of Sestrin2 in the regulation of cell growth and proliferation in cancer [9, 11, 12, 22]. A study by Budanov and Karin [9] in mouse embryonic fibroblasts (MEFs) reported that Sestrin2 inhibits cellular proliferation via reducing the transcription of cyclin D1 (a cell cycle regulator) and c-Myc (an oncogene). In addition, the study also demonstrated that knockdown of Sestrin2 renders MEF cells prone to Ras-activated oncogenic transformation. Consistent with these findings, a study in lung cancer cells by Ding et al. [11] reported that deficiency of Sestrin2 promotes migration of cancer cells in vitro and growth of xenograft tumors in vivo.

**Figure 4:**
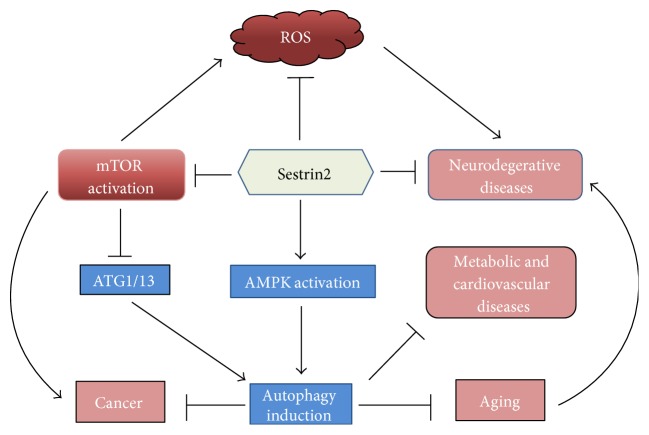
Protective effects of Sestrins in cardiovascular diseases, metabolic disorders, neurodegenerative diseases, and cancer. The antioxidant effects of Sestrins are primarily responsible for their protective effects against neurodegenerative diseases and other disease states associated with significant accumulation of ROS. Additionally, by modulating mTOR/AMPK signaling pathway by Sestrin2 indirectly represses tumor growth and activates autophagy. Consequently, autophagy activation confers a protective role of metabolic and heart diseases and aging. ATG1/13: autophagy-related protein 1 and 13; mTOR: mammalian target of rapamycin.

A recent study in nonsmall cell lung cancer (NSCLC) by Chen et al. [12] revealed that significant downregulation of Sestrin2 in the aggressive NSCLC including tumor, node, metastasis (TNM) stage and lymph node metastasis. Multivariate analyses of the results indicated an inverse relationship between the levels of Sestrin2 and the progression of NSCLC. Similarly, genetic deletion of Sestrin2 in mice was demonstrated to exhibit increased propensity to develop colon cancer [22]. Clinical evidence from colon cancer patients showing downregulation of Sestrin2 and a negative correlation between Sestrin2 levels and resistance to chemotherapy further supports the notion that Sestrin2 acts a tumor-suppressive protein and serves as a surrogate prognostic marker in multiple cancers such as NSCLC and colon cancer. The key signaling mechanisms that are modulated by Sestrin2 and how it exerts its protective effects in various disease states are shown in the Figure 4.

## 6. Conclusion

**Figure 5:**
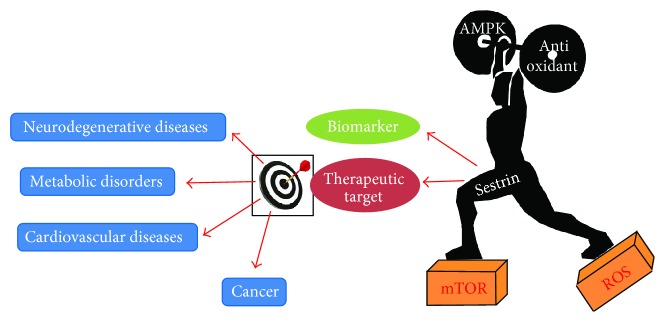
Sestrins inhibit ROS generation and mTOR activation and augment AMPK signaling and antioxidant levels in cells. These modulatory effects of Sestrins make them an attractive therapeutic target and a biomarker in various diseases such as neurodegenerative diseases, metabolic disorders, cardiovascular diseases, and cancer. AMPK: AMP-dependent protein kinase; mTOR: mammalian target of rapamycin; ROS: reactive oxygen species.

Sestrin2, a stress-responsive protein, which is mainly regulated by p53, exerts cytoprotective effects against genotoxic and oxidative stress. More importantly, Sestrin2 is now recognized as a key regulator of cell metabolism and an active contributor to cellular homeostasis in normal physiology and diseased states. Its potent antioxidant effects are shown to confer neuroprotection in neurodegenerative disorders that are closely linked to oxidative stress such as Parkinson's disease and Alzheimer's disease. As a positive regulator of AMPK and a repressor of mTORC1, Sestrins elicit protective effects in various metabolic disorders such as diabetes and obesity, cancer, cardiac hypertrophy, and atherosclerosis as shown in the Figure 5(). Thus, Sestrins demonstrate tremendous potential to serve as a favorable prognostic marker and a viable therapeutic target in various diseases.

To devise therapeutic strategies to upregulate Sestrins, it is important to decipher both the upstream and downstream pathways that underlie Sestrin's pleiotropic beneficial effects such as antioxidant effects, abrogation of hypoxic signaling and ER stress, AMPK activation/mTORC1 inhibition, autophagy activation, prosurvival effects on normal cells, and antiproliferative effects on cancer cells. Future studies using transgenic animal models with conditional, organ-specific knockout of Sestrin2 and attempts to correlate the levels of Sestrin2 (in patients' biopsy samples) with disease progression would help us to identify the biochemical pathways that are modulated by Sestrin2 in specific diseases. Furthermore, development and screening of small molecule Sestrin2 mimetics or inducers using in vitro and in vivo models would be helpful to ascertain the therapeutic potential of Sestrin2 as a drug target in various diseases.

## Figures and Tables

**Table 1:** Sestrin family of proteins, their regulators, and cellular effects.

Isoforms of Sestrin	Regulators	Effects on cell signaling
Sestrin1 (SESN1)	p53	(i) ROS inhibition via upregulation of antioxidants [1, 3, 7] (ii) Activation of AMPK pathway [1, 3, 8] (iii) Downregulation of mTOR pathway [1, 3, 6, 9] (iv) Induction of autophagy [1, 3, 7, 10] (v) Protective role against cancer [11–13], metabolic and cardiovascular diseases [14–16], and neurodegenerative disorders [17–20]
Sestrin2 (SESN2)	p53 Nrf2 AP-1	
Sestrin3 (SESN3)	AP-1 FoxO1 FoxO3	
